# Supplementary material for: Study on mechanical properties of corroded concrete columns strengthened with SMA wires
Source: PLoS One. 2023 Feb 6;18(2):e0276280. doi: 10.1371/journal.pone.0276280 (PMC9901767; doi:10.1371/journal.pone.0276280)
Supplement: Attachment — Submitted filename: Response to Reviewers.docx [file pone.0276280.s001.docx]

**Response to Reviewers**

一**、Response to the question of reviewer #1**

1. This paper inverstigates mechanical properties of reinforced concrete columns corroded in simulated seawater strengthened by shape memory alloy wires. 14 specimens were made through the test, and the actual seawater corrosion was simulated by preparing a certain concentration of artificial seawater. FEM was also conducted. This paper can be published after a professional English editor.

Response : We have asked English professionals to revise the whole manuscript.

1. **Response to the question of reviewer #2**

1. The title needs to be more concise such as Study on mechanical properties of SMA-reinforced corroded concrete columns.

Response : Thank you for your remind. The title has been changed from "Study on mechanical properties of reinforced concrete columns corroded in simulated seawater strengthened by shape memory alloy wires" to "Study on mechanical properties of corroded concrete columns strengthened with SMA wires".

2. The literature review of application of SMA wire/cable-based components (e.g., Eng. Struct., 183: 533-549, 2019; Earth. Eng. Struct. Dyn., 2021, https://doi.org/10.1002/eqe.3555.) should be referred.

Response : Thank you for your remind. The application literature review of SMA wire has been added in the introduction.

3. The manuscript needs to be polished by native speakers of English.

Response : We have polished the language of the whole manuscript.

4. The numerical simulation results together with the test results are both recommended to provide.

Response :Thank you for your remind. Numerical simulation results and test results have been supplemented.

5. The axial force vs. axial deformation of the SMA-reinforced cylinder specimen, shown in Figure 8, should be provided.

Response : This is a very detailed issue and thank you for your remind. The deformation diagram of SMA wire reinforced specimen has been added at the corresponding position.

1. **Response to the question of reviewer #3**

1. Introduction: It is a poor introduction to present why SMA can better improve the strength and the energy dissipation. It should firstly talk more about the material characteristics of the SMA and discuss how these characteristics can help to improve the capacities.

Response : Thank you for your detailed reading. It has been revised according to these suggestions in the introduction.

2. Introduction: There are different families in SMAs (Cu-Based, NiTi-based and Fe-based). Different SMAs have different properties and may behave totally different. However, this paper did not mention this.

Response : Thank you for your remind. In this paper, NiTi-SMA wire is used for reinforcement, which has been explained in the abstract and introduction. Because this paper mainly studies the reinforcement effect of SMA wire on corroded columns, and Ni-Ti SMA wire has the characteristics of hyperelasticity and good deformation ability, which can help us achieve the research purpose.

3. Section 2.1: The naming methods of the specimens were missing.

Response : Thank you for your remind. The specimen naming method has been described. For example, C1W-1 represents the first unreinforced concrete column in the first group.

4. Section 2.1: The grading of concrete is according to some Chinese standards but is lacking citations.

Response : Thank you for your detailed reading. Relevant standards have been quoted.

5. Section 2.2: There is no information about the SMA used in this study. Transformation temperatures (usually from DSC), dimensions, phase status, compositions and supplying source were not provided. So, the reliability of the paper is doubted.

Response : This is a very detailed issue and thank you for your remind. The basic information of SMA used in this test has been completed.

6. Section 2.2: How the SMA wires strengthen the concrete column is ambiguous.

Response : Thank you for your remind. The method of strengthening reinforced concrete columns with SMA wires has been described and supplemented.

7. Section 2.3: Loading protocol is missing.

Response : We have added.

8. Methods: Poor illustration of the Methods makes me difficultly understand the results.

Response : We have revised the loading method and added the loading protocol to help understand the loading method.

9. Section 3.1: There were no discussions to explain the failure modes. It looks like an experimental report rather than a scientific paper.

Response : At the beginning, what is discussed in this section is the phenomenon of the test and the final failure mode of the test piece. In this paper, there is a clear test process and test results, and the whole test is analyzed and the corresponding conclusions are drawn. And after revises, some deficiencies have also been improved.

10. Results: It is doubted how the testing programme was designed. It is apparent that reinforced concrete columns should have better capacity than the unreinforced ones no matter which material is used for reinforcement. It should be a comparison between SMA-reinforced concrete column and traditional material-reinforced concrete column. Thus, I cannot find the significance in the results part.

Response : First, the research idea proposed by reviewer is a good idea, which is also the consideration category of our original experimental design. The purpose of this paper is to study the strengthening effect of SMA wire on corroded concrete columns. The comparative analysis of materials can be studied in the next step.

11. Section 4: How was the SMA modelled? These was no material testing on SMA in this paper. How does these properties of the SMA come from? It makes the simulation results not solid.

Response : The SMA modeling process is introduced, and the SMA wire material property test has been supplemented. Thank you very much for your remind.

12. Conclusions: No solid conclusions were drawn, and the experimental problem was not well-formulated.

Response : We revised some conclusions and reached reliable conclusions from qualitative and quantitative aspects respectively. At the same time, the experimental part of the paper is also supplemented to make its conclusion more reasonable.
