# Supplementary material for: Study on mechanical properties of corroded concrete columns strengthened with SMA wires
Source: PLoS One. 2023 Feb 6;18(2):e0276280. doi: 10.1371/journal.pone.0276280 (PMC9901767; doi:10.1371/journal.pone.0276280)
Supplement: Attachment — Submitted filename: Response to Reviewers.docx [file pone.0276280.s002.docx]

### Response to Reviewers

**一、Response to the question of reviewer #2**

1. There are many typos in the current manuscript such as the unit kn in page 17 should be corrected as kN.

Response : Thank you for your careful reading. We have carefully checked the whole manuscript and revised the typos pointed out by the reviewer.

2. The last sentence of the revise manuscript is wrong and it should be rewritten.

Response : Thank you for your careful reading. We have rewritten the last sentence of the manuscript.

3. The whole revised manuscript should be checked again carefully.

Response : Thank you for your remind. We have carefully checked the whole manuscript and revised the typos.

1. **Response to the question of reviewer #3**

1. More discussions about the future research direction should be involved in the Conclusion or before the Conclusion.

Response : Thank you for your remind. We have discussed the future research direction in the conclusions and discussions.

2. A short introduction of different families of SMAs is needed in the Introduction. Following paper should be referred: Chang W-S & Araki Y. Use of shape-memory alloys in construction: a critical review. Proceedings of the Institution of Civil Engineers - Civil Engineering, 2016, 169(2), 87-95.

Response : Thank you for your remind. We have introduced different families of SMA in the introduction, and cited this literature.

3. Innovative application of SMA to devices in modern structures is suggested to be reviewed: Huang H, Chang W-S. Re-tuning an off-tuned tuned mass damper by adjusting temperature of shape memory alloy: Exposed to wind action. Structures 2020, 25, 180-189.

Response : Thank you for your remind. In the introduction, we have briefly introduced the innovative application of SMA in modern structural devices, and cited this literature.

**Note:** This change in the references is to add several references and modify the format of the references.
